# Missense Variants in *COL4A1/2* Are Associated with Cerebral Aneurysms: A Case Report and Literature Review

**DOI:** 10.3390/neurolint16010015

**Published:** 2024-02-01

**Authors:** Masahiro Uemura, Natsuki Tanaka, Shoichiro Ando, Takehiko Yanagihara, Osamu Onodera

**Affiliations:** 1 Department of Neurology, Brain Research Institute, Niigata University, Niigata 951-8585, Japanonodera@bri.niigata-u.ac.jp (O.O.); 2 Department of Neurology, Tane General Hospital, Osaka 550-0025, Japan

**Keywords:** *COL4A1*, *COL4A2*, cerebral aneurysm, small vessel disease, large vessel abnormality

## Abstract

Background: Although cerebral aneurysm (CA) is a defining complication of *COL4A1/2*-related vasculopathy, the specific factors influencing its onset remain uncertain. This study aimed to identify and analyze these factors. Methods: We described a family presenting with a novel variant of the *COL4A1* gene complicated with CA. Concurrently, an exhaustive review of previously documented patients with *COL4A1/2*-related vasculopathy was conducted by sourcing data from PubMed, Web of Science, Google Scholar, and Ichushi databases. We compared the variant types and locations between patients with CA (positive group) and those without CA (negative group). Results: This study included 53 *COL4A1/2* variants from 76 patients. Except for one start codon variant, all the identified variants in CA were missense variants. Otherwise, CA was not associated with other clinical manifestations, such as small-vessel disease or other large-vessel abnormalities. A higher frequency of missense variants (95.5% vs. 58.1%, *p* = 0.0035) was identified in the CA-positive group. Conclusions: CA development appears to necessitate qualitative alterations in *COL4A1/2*, and the underlying mechanism seems independent of small-vessel disease or other large-vessel anomalies. Our findings suggest that a meticulous evaluation of CA is necessary when missense variants in *COL4A1/2* are identified.

## 1. Introduction

Pathogenic variants are critical causes of juvenile small-vessel strokes and can manifest as large-vessel diseases beyond small-vessel disorders. Pathogenic variants in *Collagen type IV alpha 1* (*COL4A1*) and *alpha 2* (*COL4A2*) are recognized causes of small-vessel strokes, particularly intracerebral hemorrhage. Furthermore, *COL4A1/2* variants can manifest as various clinical manifestations, including retinal arterial tortuosity (RAT), kidney disease, and muscle cramps [1,2]. Cerebral aneurysm (CA) is another notable clinical feature of *COL4A1/2*-related vasculopathy that requires careful assessment and follow-up in clinical contexts. The prevalence of CA is remarkably high; previous reviews have shown that 44.4% of patients with *COL4A1/2* variants had CA, and 50.0% of them displayed multiple CAs. The most common site for CA is the internal carotid artery (ICA) [3], whereas CAs in locations other than the ICA are rare.

Several characteristic variants, such as substitution of glycine with other amino acids (AAs) in the triplet sequence of glycine (Gly-X-Y) or variants in the collagenous domain, have been reported to be associated with *COL4A1/2*-related vasculopathy [4]. Additionally, mouse models have demonstrated that clinical severity is influenced by the dosage effects of glycine substitutions in the triplet sequence [5]. Despite these insights, the genotype–phenotype correlation of CA in patients with *COL4A1/2* variants remains unclear. Large-vessel abnormalities other than CA have also been reported in patients with *COL4A1/2*-related vasculopathy [1,6]; however, the relationship between CA and those large-vessel abnormalities in *COL4A1/2* variants is yet to be clearly defined.

In this report, we present a family member carrying a novel variant in *COL4A1/2* who exhibited multiple CAs accompanied by vertebrobasilar dolichoectasia. Through an extensive literature search, we investigated the genotype–phenotype correlation among CA, small-vessel disease, and large-vessel abnormalities in patients with *COL4A1/2* variants.

## 2. Materials and Methods

- Genetic tests and exome sequencing

This study was approved by the ethics board of Niigata University (approval numbers 801, 802, and G2020-0032). Written informed consent was obtained from the patient (proband) and her mother. Genetic testing and exome sequencing (ES) were performed as described previously [7]. Briefly, genomic DNA was extracted from the proband’s blood, and Sanger sequencing analysis was performed for *NOTCH3* and *HTRA1*. After obtaining negative results for *NOTCH3* and *HTRA1*, we used ES to investigate genes associated with monogenic small-vessel disease. ES analysis was outsourced to Macrogen (Seoul, Republic of Korea). Additionally, we used Sanger’s method on the genomic DNA of the proband’s mother, using a forward primer (5′-TGGGGGCCAAACACAATTTC-3′) and a reverse primer (5′-GCGTTTCCCATCACCTCAAAC-3′).

- Literature search

We conducted a comprehensive literature search on PubMed and Web of Sciences until February 2023, focusing on publications and conference abstracts related to pathogenic variants in the *COL4A1/2* genes. Text words, phrases, and Medical Subject Headings corresponding to monogenic syndromes/diseases associated with variants in *COL4A1/2* genes were used for the search (Supplementary Materials). We also explored relevant publications using Google Scholar (https://scholar.google.jp/) and Ichushi, a Japanese publication database (https://login.jamas.or.jp/) and accessed these database on 28 February 2023. All available publications, including those that only offered abstracts without the full text, were examined. We did not restrict our search to publications in English. For publications in languages other than English or Japanese, DeepL (https://www.deepl.com/translator) or ChatGPT (https://openai.com/) was used for translation. Following the initial search, we excluded studies in which the CA status was not available.

- Classifying the variants

Variants reported in the included publications as well as those identified in our patients were analyzed using PolyPhen2, SIFT, and Provean on the VaProS website (http://p4d-info.nig.ac.jp/vapros/). Additionally, the PHRED score of the combined annotation-dependent depletion (CADD) of each variant was calculated using the CADD website (https://cadd.gs.washington.edu/). The cutoff value of the PHRED score was defined as 20 [8]. Furthermore, if variants were involved in a splicing site, additional computational analysis was performed using SpliceAI (https://spliceailookup.broadinstitute.org/) [9] and MaxEntScan (MES) (http://hollywood.mit.edu/burgelab/maxent/Xmaxentscan_scoreseq.html) accessed on 30 June 2023 [10]. The cut-off values of the spliceDelta score in SpliceAI and the %variance of MES were defined as 0.5 and 15, respectively. The %variance of MES reflects the difference between the wild-type and mutant MES, expressed as a percentage of wild-type MES [11]. A splice-site pathogenic variant is defined as pathogenic when both the %variance of MES and spliceDelta score exceed the threshold value. The frequency of each variant was determined using the Genome Aggregation Database (gnomAD) (https://gnomad.broadinstitute.org/). We also evaluated the pathogenicity of the identified variants by using ClinVar (https://www.ncbi.nlm.nih.gov/clinvar/). The pathogenicity of each variant was classified according to the American College of Medical Genetics and Genomics (ACMG) standards and guidelines [12]. Variants classified as benign or likely benign according to ACMG criteria were excluded from further analysis.

Finally, to explore the three-dimensional locations of identified mutation in the probands, we generated 3D models of using alphafold2 software (version 2.3.2) [13]. This model was employed to create images demonstrating the locations of variants specific to each group using PyMOL software, version 2.5.0 (Schrodinger, LLC, New York, NY, USA).

- Clinical assessment and comparison between CA-positive and -negative variants

To elucidate the factors associated with CA in the context of *COL4A1/2* vasculopathy, we segregated the variants into CA-positive and -negative groups. Subsequently, we compared the frequencies of the variant types and domains between the two groups. Furthermore, previous reports have demonstrated that substitutions in charged (Arg, Asp, Glu, and Lys) (https://proteinstructures.com/sequence/amino-acids/) or branched-chain AAs (Val, Leu, and Ile) [14] influence the clinical manifestations of *COL4A1/2*-related vasculopathy [4]. Therefore, we compared the frequencies of variants that were substituted with charged/branched-chain AAs.

Second, we explored whether the CA-positive phenotype correlated with other *COL4A1/2*-related phenotypes, including other large-vessel abnormalities. Patients from the literature and our own were divided into two groups according to their CA status. We compared the clinical features and imaging findings between the two groups. Signs specific to hereditary angiopathy with nephropathy, aneurysms, and muscle cramps (HANAC) syndrome (OMIM: #611773), including RAT, muscle cramps, and nephropathy (hematuria or decreased renal function), were also investigated.

- Statistics

Statistical analyses were conducted using the MATLAB R2021a software (9.10.0.01739362) (MathWorks, Inc., Natick, MA, USA). Continuous variables, such as age at stroke onset, were compared using Wilcoxon rank-sum tests because of their non-normal distribution and unequal variance. Fisher’s exact test or Chi-squared tests was used to compare frequencies of variables, such as neurological symptoms/signs. Statistical significance was defined as *p* < 0.05. In instances where information was unavailable, such data were excluded from the statistical analysis.

## 3. Results

### 3.1. Case Report

**Figure 1:**
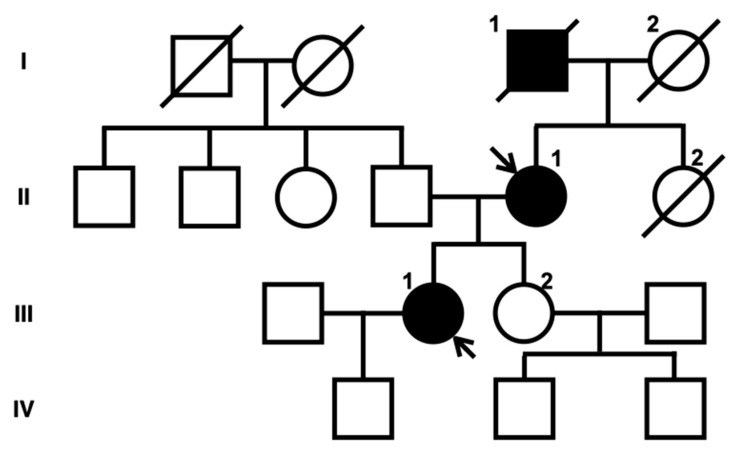
Family tree of the proband. Box and circle symbols represent male and female individuals, respectively. Filled symbols indicate individuals with a history of stroke. Diagonal lines were used to mark deceased family members. Arrows indicate the proband (III-1) and the proband’s mother (II-1).

The proband (III-1, Figure 1) had no vascular risk factors, such as hypertension, diabetes or smoking, but had migraine without aura. Upon awakening one morning at 32 years of age, she noticed difficulty in moving her right upper and lower extremities, and salivation from the right corner of her mouth. As there was no clear improvement in her symptoms, she visited Tane General Hospital the following day. Her blood pressure and heart rate were 115/44 mm Hg and 72 beats/min, respectively. Neurological examination revealed right facial palsy, dysarthria, and clumsiness of the right hand. Diffusion-weighted imaging (DWI) on the brain magnetic resonance imaging (MRI) revealed acute infarction in the left posterior limb of the internal capsule. Additionally, fluid-attenuated inversion recovery (FLAIR) images showed patchy periventricular white matter hyperintensities (WMHs) on each side. T2*-weighted images revealed multiple microbleeds (MBs). Brain MR angiography (MRA) and computed tomography angiography (CTA) revealed multiple CAs, including the lateral side of the basilar artery (BA) and the superior cerebellar artery (SCA), as well as vertebrobasilar dolichoectasia (Figure 1). The SCA aneurysm measured 10 × 10 × 12 mm.

The patient was admitted to our hospital for further evaluation and treatment with aspirin. Electrocardiography monitoring revealed no atrial fibrillation, and echocardiography showed no cardiac dysfunction. Laboratory test results, including those for protein C, S, and antiphospholipid antibodies, were negative. Urinalysis indicated hematuria, although renal function was normal. There were no remarkable ophthalmological findings, such as RAT. Her score on the Japanese edition of the Montreal Cognitive Assessment was 23, which was slightly lower than the normal threshold of 26. Cerebral angiography subsequently revealed a small ICA aneurysm at the C3 level (images not shown). The proband’s neurological symptoms gradually improved, with only mild facial hemiparesis. The SCA aneurysm was subsequently treated with coiling.

**Figure 2:**
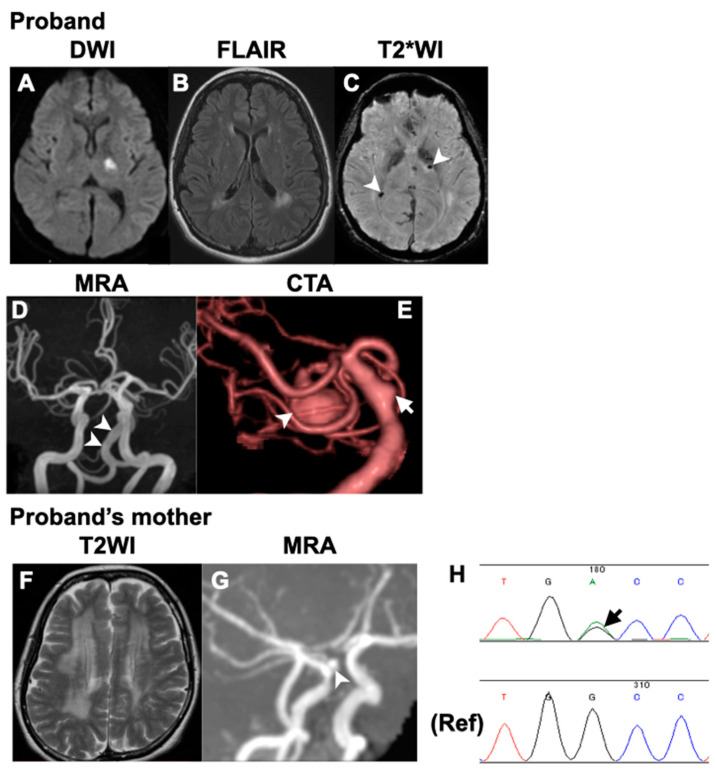
Brain imaging and the result of sequence analysis. Brain magnetic resonance images (MRIs) of the proband (**A**–**E**), including magnetic resonance angiography (MRA) (**D**) and computed tomography angiography (CTA) (**E**) as well as MRI and MRA (**F**,**G**) of the proband’s mother are presented. (**A**) Diffusion-weighted image shows acute infarction in the left posterior limb of the internal capsule. (**B**) Fluid-attenuated inversion recovery image (FLAIR) shows patchy white matter hyperintensities (WMHs) around the lateral ventricles. (**C**) T2*-weighted image displays multiple microbleeds (arrowheads). (**D**) Brain magnetic resonance angiography (MRA) shows vertebrobasilar dolichoectasia. (**E**) Computed tomography angiography (CTA) reveals a large superior cerebellar artery (SCA) aneurysm (marked by an arrowhead) and an aneurysm on the lateral side of the basilar artery (marked by an arrow). (**F**) T2 weighted image shows diffuse WMHs, and (**G**) MRA shows an aneurysm of the right ICA just above the ophthalmic artery. (**H**) Sequence analysis reveals a heterozygous variant, c.3734G>A, in *COL4A1* (marked by an arrow).

The proband’s mother (II-1, Figure 1) suffered an acute cerebellar ischemic infarction at 48 years of age, which was treated with clopidogrel. She had hypertension, but no history of diabetes, smoking or migraine. Laboratory data were not available. Brain MRI at the age of 48 revealed diffuse WMHs and a few lacunar infarctions. MRA of the brain at that time revealed a right ICA aneurysm (Figure 2).

The proband’s grandfather (I-1, Figure 1) also experienced an ischemic stroke at 54 years of age. Subsequently, he died of gastric cancer. Detailed clinical information such as medical history, laboratory data, and brain imaging was not available.

**Figure 3:**
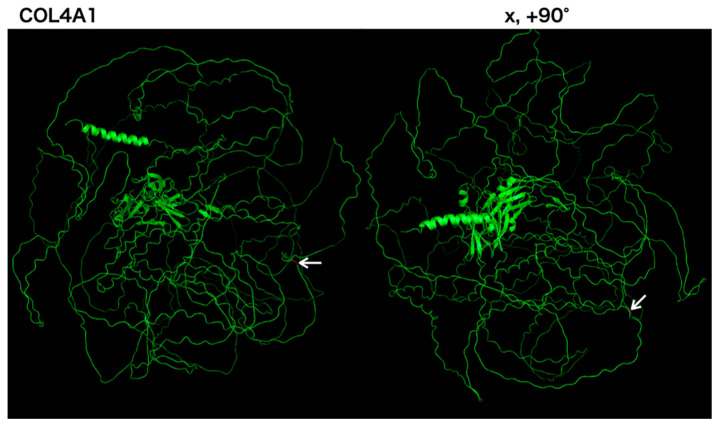
Color map of an identified missense mutation of COL4A1. Three-dimensional COL4A1 monomer structures generated by PyMol are shown. COL4A1 monomers are shown as green ribbons. The COL4A1 structure reference data were generated using the alphafold2. On the left side are images from the front view, and x, +90° indicate the degree of rotation along the x-axis, respectively. Arrows indicate Gly1245 in COL4A1.

Owing to the young onset of stroke and family history, we suspected a monogenic stroke. After obtaining consent for genetic testing from the proband and her mother, we performed a sequence analysis. ES revealed a novel heterozygous variant in exon 42 of the *COL4A1* gene (c.3734G>A, p.Gly1245Asp). The p.Gly1245 site is located within the glycine triplet sequence (Gly-X-Y) of the collagenous domain of *COL4A1* (Figure 3). Using Sanger sequencing, we detected the same variant in the proband’s mother (Figure 2).

### 3.2. Literature Review

We conducted a comprehensive review of previous publications on *COL4A1/2* variants. Initially, our search identified 470 publications from PubMed and 452 from Web of Science that matched our search terms. After eliminating duplicate publications, we added 6 publications from Ichushi and 13 from Google Scholar to our pool of resources. These publications were screened by title or abstract, and 444 publications were excluded. Subsequently, full-text screening was conducted, and 55 publications were ultimately included in our study (Supplementary Figure S1).

From these publications, 55 variants were identified. These variants were classified according to the ACMG criteria and three were found to be likely benign (p.Gln1150Lys, p.Glu1123Gly, and p.Ala1690Thr in *COL4A2*) [15,16,17] (Supplementary Table S3). These three variants and their corresponding publications were excluded from further analysis. Ultimately, our study included 74 patients, 52 variants from 52 publications, and two of our patients.

The variants included in this study are summarized in Supplementary Tables S1 and S2. Of the 53 variants, 39 were missense variants. Other variants included three duplication/copy number variants (CNVs), two frameshift variants, four splice site variants, one start codon variant, and four point variants in the 3′-untranslated region (UTR). All four splice site variants were located at canonical splice sites, and three of the four variants were predicted to cause in-frame variants due to exon skipping [18,19,20,21].

- Characteristics of CA-positive Variants

First, we divided the included variants according to whether CA had been previously reported (Table 1 and Supplementary Table S4). Among 53 variants, CA was detected in 22. Among the variants in *COL4A1*, except for one start codon mutation, no variants other than missense variants were found in the CA-positive group. As compared with the CA-negative group, the frequency of missense variants (94.4% vs. 59.1%, *p* = 0.0126) and variants substituted with charged/branched-chain AAs in *COL4A1* were significantly higher in the CA-positive group than in the negative group (77.8% vs. 40.9%, *p* = 0.0267), whereas glycine substitution variants in the triplet sequence (Gly-X-Y) was similar between the two groups (72.2% vs. 54.5%, *p* = 0.3319).

Although there were no significant differences in the frequency of variant types or domains due to the small sample size of the variants in *COL4A2*, all four CA-positive variants were located at the glycine site of the triplet sequence, in addition to charged/branched-chain AAs substitutions.

In combination with variants in *COL4A1/2* and duplication/CNV, the frequencies of missense variants (95.5% vs. 58.1%, *p* = 0.0035), glycine substitution variants in the glycine triplet sequence (77.3% vs. 48.4%, *p* = 0.0475), variants substituted by charged/branched-chain AAs (81.8% vs. 38.7%, *p* = 0.0022), and variants in the triple-helical domain (90.9% vs. 64.5%, *p* = 0.0497) were significantly higher in the CA-positive group than in the negative group.

**Figure 4:**
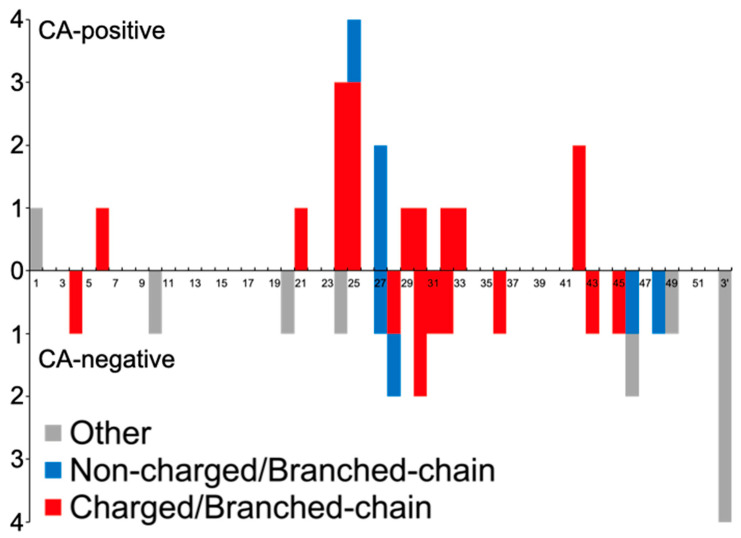
Distribution of variants in *COL4A1*. In the graph, the exon positions of *COL4A1* are presented along the horizontal axis, and the number of mutations is depicted on the vertical axis. Missense mutations in *COL4A1* that give rise to charged/branch-chain amino acid substitutions are highlighted in red. These missense mutations are particularly noteworthy as our findings suggest their relevance to cerebral aneurysms (CA). Mutations that resulted in substitutions other than the charged/branch chain amino acids are denoted in blue, while other mutation types are indicated in gray. For splice site mutations, the exons predicted to undergo skipping are shown. The 3′ untranslated region (3′ UTR) is denoted as 3′.

Next, we demonstrated the distribution of variants resulting in substitution with charged/branched-chain AAs in *COL4A1* (Figure 4). Variants in the CA-positive group were primarily concentrated in exons 24 and 25, and then in exons 27 and 42 of *COL4A1*.

- Characteristics of CAs in patients with *COL4A1/2* variants

The patients were categorized into positive and negative groups according to the presence of CA. Among the 76 patients with *COL4A1/2* variants, 25 had CA. The age at stroke onset and frequency of family or medical history of stroke were similar in both groups. No patients with subarachnoid hemorrhage (SAH) were found in the CA-positive group, whereas one patient in the CA-negative group had a history of SAH resulting from traumatic injury [6]. Geographic distribution was similar between the two groups. In addition, imaging findings of small-vessel disease were equally similar in the two groups. The frequencies of other clinical findings such as RAT, muscle cramps, and nephropathy were also similar. However, the frequency of migraines was significantly higher in the CA-positive group (Supplementary Table S5). The proportion of modalities used to evaluate cerebral vessels was as follows for the CA-positive and the CA-negative group, respectively: MRA was 68.0% and 72.5%, CTA was 20.0% and 13.7%, and cerebral angiography was 28.0% and 15.7%.

The ICA is the most affected artery, with 86.4% of patients exhibiting ICA aneurysms. In addition to ICA, other CA are rare. A single instance of aneurysm in the middle cerebral artery (MCA) [6], anterior communicating artery (Acom) [22], posterior communicating artery (Pcom) [23], and superior cerebellar artery (SCA) were observed, and two patients had basilar artery (BA) aneurysms. The BA aneurysm was located at the top of the BA in one patient [24], whereas our proband had a CA on the lateral side of the BA in addition to SCA. All patients with CA in arteries other than the ICA carried missense variants in *COL4A1*, and four out of five of these variants involved substitutions of glycine with charged/branched-chain AAs in the glycine triplet sequence (Table 2). The prevalence of multiple CA was 54.5%. All identified variants in cases of multiple CA involved charged/branched-chain AA substitutions and glycine substitutions in the glycine triplet sequence (Gly498Val, Gly501Asp, Gly525Leu, Gly528Glu, Gly755Arg, Gly835Glu, Gly888Arg, Gly1245Asp in *COL4A1*, and Gly702Asp and Gly1389Arg in *COL4A2*), except for Pro116Leu in *COL4A1*. Notably, 10 of the 12 patients had multiple ICA aneurysms.

## 4. Discussion

In this study, we presented two patients carrying a novel *COL4A1* variant, and one of them had multiple CAs, including an SCA aneurysm. Thus, we extended our investigation to a review of previous publications on *COL4A1/2* variants, and demonstrated that CAs outside the ICA are rare. Although there have been no reports of SAH caused by aneurysmal rupture among patients with *COL4A1/2* variants, we chose to treat the SCA aneurysm in the proband because the risk of rupture was presumed to be high [25].

This review presents several novel findings. First, all mutations except for a start codon mutation, in the CA group are missense variants. In addition, the frequency of missense mutations with charged/branched side-chain AA substitution was higher in CA-positive patients. No patients with frameshift, splice site, duplication/CNV, or 3′-UTR variants had CAs. To date, the role of *COL4A1/2* in aneurysm development remains unclear. A recent report showed that loss of *COL4A1* expression is associated with the development of abdominal aneurysms [26]. However, our results suggest that quantitative changes are not sufficient and that qualitative changes of COL4A1/2 proteins are necessary for CA development.

COL4A1/2 are network-forming extracellular proteins that interact with several extracellular matrix proteins such as integrins and laminins. Variants in HANAC syndrome are clustered in amino acids 498–528, which are encoded by exons 24 and 25 of *COL4A1*. However, Gly1245Asp was located outside the clustering sites [27], which suggests that the pathomechanism of Gly1245Asp may differ from the mutations located within clustering sites. A recent report showed that a soluble form of the COL4A1 protein, induced by X-box binding protein 1, promotes the migration of vascular progenitor cells to injured vessels [28]. This soluble form of COL4A1 is a transcript variant generated by joining the front part of exon 4 with the rear part of exon 42 and contains the Gly1245 sequences of *COL4A1*. Therefore, this variant may cause functional impairment of the soluble form of *COL4A1*, resulting in insufficient vessel repair.

Next, we demonstrated that CA could develop irrespective of the status of small-vessel disease or other large-vessel abnormalities. We investigated clinical findings or signs related to small-vessel disease, including stroke, RAT, or nephropathy, and found that the frequency of these clinical findings was similar between the two groups. Similarly, the frequency of large-vessel abnormalities other than CA was comparable between the two groups. These results suggest that the mechanism of aneurysm formation is distinct from that of small-vessel disease or other large-vessel abnormalities. Although various factors, such as inflammatory response and shear stress, can contribute to CA formation, the precise mechanism remains elusive. Generally, CAs preferentially develop in the MCA, Acom, Pcom, and BA because of high local shear stress [29]. However, the preferential site of CA in *COL4A1/2*-related vasculopathy differs from that in general CAs. Our review revealed that more than 80% of patients had ICA aneurysms, and 10 out of 25 patients had multiple ICA aneurysms. These characteristic predispositions among patients with *COL4A1/2* variants suggest that the underlying mechanisms beyond shear stress play a significant role in CA development.

This study has several limitations. First, the extracted publications often provided insufficient descriptions of *COL4A1/2*-related symptoms or signs, leading to several instances of missing data, making it difficult to interpret clinical and imaging findings. Second, CA was defined as the presence of an aneurysm, regardless of its size or shape. Finally, as a literature review, our study is subject to several biases, such as selection bias or reporting bias.

## 5. Conclusions

Here, we report two patients of a family with a novel *COL4A1* variant. Furthermore, our results suggested that CA in patients with *COL4A1/2*-related vasculopathy is caused by missense variants. CAs can develop irrespective of other vasculopathies, such as small-vessel stroke or other large-vessel complications. Our findings underscore the need for the careful evaluation of CAs if missense variants in *COL4A1/2* are identified.

## Figures and Tables

**Table 1:** Comparison between CA-positive and negative variants. G-M-Y indicates that the variant is located at the amino acid position after the glycine residue in the glycine triplet sequence. G-X-M indicates that the variant is located at amino acid position two after the glycine residue in the glycine triplet sequence. M-X-Y indicates that the variant is located at the glycine position in the glycine triplet sequence. The UTR represents the untranslated region. NC1 represents the non-collagenous domain. AAs are amino acids. CNV is an abbreviation for copy number variant.

Mutation	Positive	Negative	*p*-Value
*COL4A1* + *COL4A2* + Duplications/CNV	*n* = 22	*n* = 31	
Variant type			
Missense	21 (95.5)	18 (58.1)	0.0035
G-M-Y	1 (4.5)	1 (3.2)	1
G-X-M	2 (9.1)	0 (0)	0.16763
M-X-Y	17 (77.3)	15 (48.4)	0.0475
Charged/Branched-chain AAs	18 (81.8)	12 (38.7)	0.0022
Other than missense variants			
3′UTR	0 (0)	4 (12.9)	0.1324
Duplication/CNV	0 (0)	3 (9.7)	0.2576
Frameshift	0 (0)	2 (6.5)	0.5051
Splice Site	0 (0)	4 (12.9)	0.1324
Start Codon	1 (4.5)	0 (0)	0.4151
Domain			
7S	1 (4.5)	1 (3.2)	1
NC1	0 (0)	3 (9.7)	0.2576
Signal	1 (4.5)	0 (0)	0.4151
Triple-helical	20 (90.9)	20 (64.5)	0.0497

**Table 2:** Variants found in patients with CA outside the ICA. CA indicates cerebral aneurysm; ICA, internal carotid artery; MCA, middle cerebral artery; Acom, anterior communicating artery; Pcom, posterior communicating artery; SCA, superior cerebellar artery.

	CA-Positive	Ref.
MCA	*COL4A1*, Gly498Val	[6]
Acom	*COL4A1*, Gly417Arg	[22]
Pcom	*COL4A1*, Pro648Ala	[23]
BA	*COL4A1*, Gly1245Asp *COL4A1*, Gly720Asp	ours [24]
SCA	*COL4A1*, Gly1245Asp	ours

## Data Availability

For appropriate reasons, the data supporting this research will be made available by the corresponding author.

## Supplementary Materials

The supplemental information can be downloaded at: https://www.mdpi.com/article/10.3390/neurolint16010015/s1. References [1,2,6,18,19,20,22,23,24,30,31,32,33,34,35,36,37,38,39,40,41,42,43,44,45,46,47,48,49,50,51,52,53,54,55,56,57,58,59,60,61,62,63,64,65,66,67,68,69,70,71,72] are cited in the supplementary materials.
