# Supplementary material for: Missense Variants in COL4A1/2 Are Associated with Cerebral Aneurysms: A Case Report and Literature Review
Source: Neurol Int. 2024 Feb 1;16(1):226–38. doi: 10.3390/neurolint16010015 (PMC10892350; doi:10.3390/neurolint16010015)

neurology international

MDPI

SUPPLEMENTARY Materials

Literature search

#1.

(“Collagen Type IV"[Mesh] AND “alpha?1”[All Fields]) OR (“Collagen Type IV"[Mesh] AND “alpha?2”[All Fields]) OR “Collagen Type IV alpha 1”[All Fields] OR “Collagen Type IV alpha 2”[All Fields] OR “COL4A1 protein, human”[Supplementary Concept] OR “COL4A2 protein, human”[Supplementary Concept] OR COL4A1[All Fields] OR COL4A2[All Fields]

#2.

Stroke[Mesh] OR Stroke[All Fields] OR “Cerebral Small Vessel Diseases”[Mesh] OR “Cerebral Small Vessel Diseases”[All Fields] OR (Cerebral[All Fields] AND Small[All Fields] AND Vessel[All Fields] AND Diseases[All Fields]) OR CSVD[All Fields] OR SVD[All Fields] OR Hemorrhage[Mesh] OR "Hemorrhagic"[All Fields] OR Leukoencephalopathies[Mesh] OR Leukoencephalopathies [All Fields] OR Leukoencephalopathy[All Fields] OR “cerebral aneurysm"[All Fields] OR "intracranial aneurysm"[All Fields] OR (cerebral[All Fields] AND aneurysm[All Fields]) OR (intracranial[All Fields] AND aneurysm[All Fields]) OR "intracerebral hemorrhage"[All Fields] OR "intracerebral haemorrhage"[All Fields] OR (intracerebral [All Fields] AND hemorrhage [All Fields]) OR (intracerebral [All Fields] AND haemorrhage [All Fields]) OR "intracranial hemorrhage"[All Fields] OR "intracranial haemorrhage"[All Fields] OR (intracranial [All Fields] AND hemorrhage [All Fields]) OR (intracranial [All Fields] AND haemorrhage [All Fields]) OR "cerebral hemorrhage"[All Fields] OR "cerebral haemorrhage"[All Fields] OR Microbleeds[All Fields] OR (cerebral [All Fields] AND hemorrhage [All Fields]) OR (cerebral [All Fields] AND haemorrhage [All Fields]) OR Dementia[Mesh] OR Dementia[All Fields] OR "Dementia, Vascular"[Mesh] OR "Vascular Dementia"[All Fields] OR (Vascular[All Fields] AND Dementia[All Fields]) OR "Dementia, Multi-Infarct"[Mesh] OR "Cognitive Dysfunction"[Mesh] OR "Cognitive Dysfunction"[All Fields] OR "Cognitive Impairment"[All Fields] OR (Cognitive[All Fields] AND Impairment[All Fields])

#3.

“autosomal dominant”[All Fields] OR “autosomal recessive”[All Fields] OR familial[All Fields] OR hereditary[All Fields] OR Hematuria[Mesh] OR Hematuria[All Fields] OR “artery tortuosity”[All Fields] OR “arterial tortuosity”[All Fields] OR Porencephaly[Mesh] OR Porencephaly[All Fields]

#4.

"Angiopathy, Hereditary, With Nephropathy, Aneurysms, And Muscle Cramps"[Supplementary Concept] OR HANAC[All Fields] OR (Angiopathy[All Fields] AND Hereditary[All Fields] AND Nephropathy[All Fields] AND Aneurysms[All Fields] AND "Muscle Cramps"[All Fields]) OR PADMAL [All Fields] OR (pontine[All Fields] AND autosomal[All Fields] AND dominant[All Fields] AND microangiopathy[All Fields] AND leukoencephalopathy[All Fields]) OR ("multi-infarct"[All Fields] AND dementia [All Fields] AND Swedish [All Fields]) OR hMID[All Fields] OR HEMID[All Fields]

#1 AND (#2 OR #3) OR #4

neurology international

MDPI

Supplementary Table S1. The results of ACMG classification of excluded mutations

Mutations	rsID	ClinVar	PVS	PS	PM	PP	BS	BP	ACMG criteria
COL4A2									
c.3368A>G, p.Glu1123Gly	rs117412802	Benign/Likely Benign	-	-	1, 2	3	4	4, 6	Likely benign
c.3448C>A, p.Gln1150Lys	NA	Benign/Likely Benign	-	-	1, 2	3, 4	-	4, 6	Likely benign
c.5068G>A, p.Ala1690Thr	rs201105747	Benign/Likely Benign	-	-	2	3	-	4, 6	Likely benign

NA indicates not available.

Supplementary Table S2. Mutations included in this study

Mutations	Mutation Type	Domain	Triplet	Exon, Intron	Allele frequency	rsID	ACMG Criteria	Aneurysm	Ref
Duplication/CNV	-	-	-	-	-	NA	Uncertain significance	Negative	[30]
Duplication/CNV	-	-	-	-	-	NA	Uncertain significance	Negative	[31]
Duplication/CNV	-	-	-	-	-	NA	Uncertain significance	Negative	[32]
				COL4A1					
c.1A>T	Start Codon	Signal	-	1	-	NA	Pathogenic	Positive	[33]
c.236G>T, p.Gly79Val	Missense	7S	M-X-Y	4	-	NA	Uncertain significance	Negative	[34]
c.347C>T, p.Pro116Leu	Missense	7S	G-M-Y	6	0.00001972	rs538816765	Uncertain significance	Positive	[35,36]
c.553-2A>G	Splice Site	Triple	-	Intron 9	-	NA	Pathogenic	Negative	[19]
c.1120+2_1120+8del	Splice Site	Triple	-	Intron 20	-	NA	Pathogenic	Negative	[18]
c.1249G>C, p.Gly417Arg	Missense	Triple	M-X-Y	21	-	NA	Pathogenic	Positive	[22]
c.1493G>T, p.Gly498Val	Missense	Triple	M-X-Y	24	-	NA	Pathogenic	Positive	[1,6]
c.1502G>A, p.Gly501Asp	Missense	Triple	M-X-Y	24	-	NA	Pathogenic	Positive	[37]
c.1528G>A, p.Gly510Arg	Missense	Triple	M-X-Y	24	-	NA	Pathogenic	Positive	[38–40]
c.1537-2delA	Splice Site	Triple	-	Intron 24	-	NA	Pathogenic	Negative	[41]
p.Arg538Trp	Missense	Triple	Other	25	-	NA	Uncertain significance	Positive	[42]
c.1555G>A, p.Gly519Arg	Missense	Triple	M-X-Y	25	-	NA	Pathogenic	Positive	[1,6]
c.1573GG>TT, p.Gly525Leu	Missense	Triple	M-X-Y	25	-	NA	Pathogenic	Positive	[40]
c.1583G>A, p.Gly528Glu	Missense	Triple	M-X-Y	25	-	NA	Pathogenic	Positive	[1,6]
c.1937G>C, p.Gly646Ala	Missense	Triple	M-X-Y	27	0.000006573	rs532972509	Pathogenic	Negative	[43]
c.1942C>G, p.Pro648Ala	Missense	Triple	G-X-M	27	0.000006569	rs1413099763	Likely pathogenic	Positive	[23]
c.1961C>A, p.Prp654Gln	Missense	Triple	G-X-M	27	0.00001314	rs758315813	Likely pathogenic	Positive	[44]
c.2063G>A, p.Gly688Asp	Missense	Triple	M-X-Y	28	-	NA	Likely pathogenic	Negative	[45]
c.2086G>A, p.Gly696Ser	Missense	Triple	M-X-Y	28	-	NA	Pathogenic	Negative	[46]
c.2159G>A, p.Gly720Asp	Missense	Triple	M-X-Y	29	-	NA	Pathogenic	Positive	[24,47]

c.2263G>A, p.Gly755Arg	Missense	Triple	M-X-Y	30	-	NA	Pathogenic	Positive	[48,49]
c.2317G>A, p.Gly773Arg	Missense	Triple	M-X-Y	30	-	NA	Pathogenic	Negative	[50]
c.2327G>T, p.Gly776Val	Missense	Triple	M-X-Y	30	-	NA	Pathogenic	Negative	[51]
c.2413G>A, p.Gly805Arg	Missense	Triple	M-X-Y	31	-	NA	Pathogenic	Negative	[52]
c.2494G>A, p.Gly832Arg	Missense	Triple	M-X-Y	32	-	NA	Pathogenic	Negative	[53]
c.2504G>A, p.Gly835Glu	Missense	Triple	M-X-Y	32	-	NA	Pathogenic	Positive	[54]
c.2662G>C, p.Gly888Arg	Missense	Triple	M-X-Y	33	-	NA	Pathogenic	Positive	[22]
c.2969G>T, p.Gly990Val	Missense	Triple	M-X-Y	36	-	NA	Likely pathogenic	Negative	[55]
c.3715G>A, p.Gly1239Arg	Missense	Triple	M-X-Y	42	-	NA	Likely pathogenic	Positive	[56]
c.3734G>A, p.Gly1245Asp	Missense	Triple	M-X-Y	42	-	NA	Pathogenic	Positive	Ours
c.3797G>T, p.Gly1266Val	Missense	Triple	M-X-Y	43	-	NA	Likely pathogenic	Negative	[57]
c.3976G>A, p.Gly1326Arg	Missense	Triple	M-X-Y	45	-	NA	Pathogenic	Negative	[58]
c.4031G>C, p.Gly1344Ala	Missense	Triple	M-X-Y	46	-	NA	Likely pathogenic	Negative	[59]
c.4150+1G>T	Splice Site	Triple	-	Intron 46	-	NA	Pathogenic	Negative	[20]
c.4380T>G, p.Cys1460Trp	Missense	NC1	Other	48	-	NA	Uncertain significance	Negative	[60]
c.4611_4612insG, p.Thr1537fs	Frameshift	NC1	Other	49	-	NA	Pathogenic	Negative	[61]
c.*32G>A	3'UTR	-	Other	3'UTR	-	NA	Likely pathogenic	Negative	[62]
c.*32G>T	3'UTR	-	Other	3'UTR	-	NA	Likely pathogenic	Negative	[63]
c.*34G>T	3'UTR	-	Other	3'UTR	-	NA	Uncertain significance	Negative	[64]
c.*35C>A	3'UTR	-	Other	3'UTR	-	NA	Likely pathogenic	Negative	[65]
				COL4A2					
c.1837G>A, p.Gly613Ser	Missense	Triple	M-X-Y	24	-	NA	Pathogenic	Negative	[66]
c.2105G>A, p.Gly702Asp	Missense	Triple	M-X-Y	28	-	NA	Pathogenic	Positive	[67]
c.2572A>G, p.Ile858Val	Missense	Triple	G-M-Y	29	-	NA	Uncertain significance	Negative	[68]
c.2821G>A, p.Gly941Arg	Missense	Triple	M-X-Y	32	-	NA	Pathogenic	Positive	[69]
c.2972G>A, p.Gly991Glu	Missense	Triple	M-X-Y	32	-	NA	Pathogenic	Positive	[70]
c.3110G>A, p.Gly1037Glu	Missense	Triple	M-X-Y	33	-	NA	Pathogenic	Negative	[2]

c.3206delC	Frameshift	Triple	Other	34	-	NA	Pathogenic	Negative	[71]
c.3455G>A, p.Gly1152Asp	Missense	Triple	M-X-Y	37	-	NA	Pathogenic	Negative	[2]
c.4165G>A, p.Gly1389Arg	Missense	Triple	M-X-Y	44	0.000006574	NA	Pathogenic	Positive	[71]
p.Ala1534Ser	Missense	NC1	Other	46	0.00001314	rs760399386	Uncertain significance	Negative	[72]

CNV, copy number variant; UTR, untranslated region; NC1, non-collagenous domain; NA, not available. G-M-Y indicates that the mutation is located at the amino acid position after the glycine residue in the glycine triplet sequence. G-X-M indicates that the mutation is located at amino acid position two after the glycine residue in the glycine triplet sequence. M-X-Y indicates that the mutation is located at the glycine position in the glycine triplet sequence.

Supplementary Table S3. The results of in silico analysis and ACMG classification of included mutations in this study

Mutations	ClinVar	delta score of SpliceAI				MaxEntScan, %Variance	CADD, PHRED	PolyPhen2	SIFT	Provean	ACMG				Ref
		Acceptor Loss	Donor Loss	Acceptor Gain	Donor Gain						PVS	PS	PM	PP	
Duplication/CNV	NA	-	-	-	-	-	-	NA	NA	NA	-	-	2	4	[30]
Duplication/CNV	NA	-	-	-	-	-	-	NA	NA	NA	-	-	2, 6	4	[31]
Duplication/CNV	NA	-	-	-	-	-	-	NA	NA	NA	-	-	2	4	[32]
COL4A1															
c.1A>T	Pathogenic	-	-	-	-	-	23.2	NA	NA	NA	1	-	2	5	[33]
c.236G>T, p.Gly79Val	NA	0	0	0	0	4.8	33.0	Probably	Deleterious	Deleterious	-	-	2	3, 4	[34]
c.347C>T, p.Pro116Leu	Uncertain Significance	-	-	-	-	-	24.1	Probably	Tolerated	Deleterious	-	-	2	3	[35,36]
c.553-2A>G	NA	1	0	0.29	0	79.6	32.0	NA	NA	NA	1	2	1, 2	3, 4	[19]
c.1120+2_1120+8del	NA	0.13	0.99	0	0.01	176.9	23.5	NA	NA	NA	1	-	1, 2	1, 3, 4	[18]
c.1249G>C, p.Gly417Arg	NA	-	-	-	-	-	21.6	benign	Tolerated	Deleterious	-	3	1, 2	3, 4	[22]
c.1493G>T, p.Gly498Val	Pathogenic	-	-	-	-	-	25.6	Probably	Deleterious	Deleterious	-	3	1, 2	3, 4, 5	[1,6]
c.1502G>A, p.Gly501Asp	Pathogenic/Likely Pathogenic	-	-	-	-	-	25.8	Probably	Deleterious	Deleterious	-	3	1, 2	3, 4, 5	[37]
c.1528G>A, p.Gly510Arg	Pathogenic/Likely Pathogenic	-	-	-	-	-	24.2	Probably	Deleterious	Deleterious	-	3	1, 2	1, 3, 4, 5	[38–40]
c.1537-2delA	NA	0.98	0	0.14	0.02	163.9	29.5	NA	NA	NA	1	2	1, 2	3, 4	[41]
p.Arg538Trp	Uncertain Significance	-	-	-	-	-	23.9	Probably	Tolerated	Deleterious	-	-	1, 2	3	[42]
c.1555G>A, p.Gly519Arg	Pathogenic	-	-	-	-	-	26.6	Probably	Deleterious	Deleterious	-	3	1, 2	3, 4, 5	[1,6]
c.1573GG>TT, p.Gly525Leu	Pathogenic	-	-	-	-	-	26	Probably	Deleterious	Deleterious	-	3	1, 2	3, 4, 5	[40]
c.1583G>A, p.Gly528Glu	Pathogenic	-	-	-	-	-	27.2	Probably	Deleterious	Deleterious	-	3	1, 2	3, 4, 5	[1,6]
c.1937G>C, p.Gly646Ala	NA	-	-	-	-	-	22.7	Probably	Tolerated	Deleterious	-	3	1, 2	3, 4	[43]
c.1942C>G, p.Pro648Ala	NA	-	-	-	-	-	22.6	Probably	Tolerated	Deleterious	-	-	1, 2	3, 4	[23]
c.1961C>A, p.Prp654Gln	NA	-	-	-	-	-	19.7	Possibly	Deleterious	Deleterious	-	-	1, 2	3, 4	[44]
c.2063G>A, p.Gly688Asp	NA	-	-	-	-	-	23.7	Probably	Tolerated	Neutral	-	3	1, 2	3	[45]
c.2086G>A, p.Gly696Ser	Pathogenic	-	-	-	-	-	27	Probably	Deleterious	Deleterious	-	2, 3	1, 2	3, 4, 5	[46]
c.2159G>A, p.Gly720Asp	Pathogenic/Likely Pathogenic	-	-	-	-	-	25.5	Probably	Deleterious	Deleterious	-	3	1, 2	3, 4, 5	[24,47]
c.2263G>A, p.Gly755Arg	Pathogenic	-	-	-	-	-	24.3	Probably	Deleterious	Deleterious	-	2, 3	1, 2	3, 4, 5	[48,49]

c.2317G>A, p.Gly773Arg	Pathogenic	-	-	-	-	-	24.4	Probably	Deleterious	Deleterious	-	2, 3	1, 2	3, 4, 5	[50]
c.2327G>T, p.Gly776Val	Likely Pathogenic	-	-	-	-	-	25.8	Probably	Deleterious	Deleterious	-	3	1, 2	3, 4, 5	[51]
c.2413G>A, p.Gly805Arg	Likely Pathogenic	-	-	-	-	-	25.5	Probably	Deleterious	Deleterious	-	3	1, 2	3, 4, 5	[52]
c.2494G>A, p.Gly832Arg	Pathogenic/Likely Pathogenic	-	-	-	-	-	27.5	Possibly	Deleterious	Deleterious	-	2, 3	1, 2	3, 4, 5	[53]
c.2504G>A, p.Gly835Glu	NA	-	-	-	-	-	26.5	benign	Deleterious	Deleterious	-	2, 3	1, 2	3, 4	[54]
c.2662G>C, p.Gly888Arg	Pathogenic/Likely Pathogenic	-	-	-	-	-	25.9	Possibly	Deleterious	Deleterious	-	2, 3	1, 2	3, 5	[22]
c.2969G>T, p.Gly990Val	Likely Pathogenic	0.06	0	0.01	0	33.0	33	Probably	Deleterious	Deleterious	-	-	1, 2, 5	3, 4, 5	[55]
c.3715G>A, p.Gly1239Arg	Pathogenic	-	-	-	-	-	26.4	Probably	Deleterious	Deleterious	-	-	1, 2	3, 4, 5	[56]
c.3734G>A, p.Gly1245Asp	NA	-	-	-	-	-	26.3	Probably	Deleterious	Deleterious	-	3	1, 2	3, 4	Ours
c.3797G>T, p.Gly1266Val	NA	-	-	-	-	-	24.1	Probably	Deleterious	Deleterious	-	3	1, 2	3	[57]
c.3976G>A, p.Gly1326Arg	Pathogenic	-	-	-	-	-	26.9	Probably	Deleterious	Deleterious	-	2, 3	1, 2	3, 5	[58]
c.4031G>C, p.Gly1344Ala	NA	-	-	-	-	-	24.9	Possibly	Deleterious	Deleterious	-	3	1, 2	3	[59]
c.4150+1G>T	NA	0	0.99	0	0.05	97.9	33	NA	NA	NA	1	2	1, 2	3, 4	[20]
c.4380T>G, p.Cys1460Trp	NA	-	-	-	-	-	25.7	Probably	Deleterious	Deleterious	-	-	2	3	[60]
c.4611_4612insG, p.Thr1537fs	NA	-	-	-	-	-	33	NA	NA	NA	1	-	2	4	[61]
c.*32G>A	Likely Pathogenic	-	-	-	-	-	19.2	NA	NA	NA	-	3	2	4, 5	[62]
c.*32G>T	Pathogenic	-	-	-	-	-	18.7	NA	NA	NA	-	3	2	4, 5	[63]
c.*34G>T	NA	-	-	-	-	-	18.9	NA	NA	NA	-	-	2	4, 5	[64]
c.*35C>A	Pathogenic	-	-	-	-	-	19	NA	NA	NA	-	3	2	4, 5	[65]
COL4A2															
c.1837G>A, p.Gly613Ser	NA	-	-	-	-	-	24.7	Probably	Deleterious	Deleterious	-	3	1, 2	3, 4	[66]
c.2105G>A, p.Gly702Asp	NA	-	-	-	-	-	27.5	Probably	Tolerated	Deleterious	-	2, 3	1, 2	3, 4	[67]
c.2572A>G, p.Ile858Val	NA	-	-	-	-	-	1.0	benign	Tolerated	Neutral	-	-	1, 2	-	[68]
c.2821G>A, p.Gly941Arg	NA	-	-	-	-	-	26.5	Probably	Deleterious	Deleterious	-	2, 3	1	3, 4	[69]
c.2972G>A, p.Gly991Glu	NA	-	-	-	-	-	24.8	Probably	Deleterious	Deleterious	-	3	1, 2	3, 4	[70]
c.3110G>A, p.Gly1037Glu	Pathogenic	-	-	-	-	-	25.7	Probably	Deleterious	Deleterious	-	2, 3	1, 2	3, 4, 5	[2]
c.3206delC	NA	-	-	-	-	-	33	NA	NA	NA	1		1, 2	4	[71]
c.3455G>A, p.Gly1152Asp	Pathogenic	0.01	0	0.03	0	4.8	29.4	Probably	Deleterious	Deleterious	-	3	1, 2	3, 4, 5	[2]
c.4165G>A, p.Gly1389Arg	Likely Pathogenic	-	-	-	-	-	25.5	Probably	Deleterious	Deleterious	-	3	1, 2	3, 4, 5	[71]

p.Ala1534Ser	NA	-	-	-	-	-	25.7	Probably	Tolerated	Deleterious	-	-	2	3, 4	[72]

CNV, copy number variants; CADD, combined annotation-dependent depletion; probably, probably damaging; possibly damaging; NA, not available.

Supplementary Table S4. Comparison between CA positive and negative mutations

	Positive	Negative	p-value
COL4A1	n = 18	n = 22	-
Mutations			
Missense	17 (94.4)	13 (59.1)	0.0126
G-M-Y	1 (5.6)	0 (0)	0.45
G-X-M	2 (11.1)	0 (0)	0.1962
M-X-Y	13 (72.2)	12 (54.5)	0.3319
Charged/Branched-chain AAs	14 (77.8)	9 (40.9)	0.0267
Other than missense mutations	1 (5.6)	9 (40.9)	0.0126
3'UTR	0 (0)	4 (18.2)	0.1135
Frameshift	0 (0)	1 (4.5)	1
Splice Site	0 (0)	4 (18.2)	0.1135
Start Codon	1 (5.6)	0 (0)	0.45
Domain			
7S	1 (5.6)	1 (4.5)	1
NC1	0 (0)	2 (9.1)	0.4923
Signal	1 (5.6)	0 (0)	0.45
Triple-helical	16 (88.9)	15 (68.2)	0.1489
COL4A2	n = 4	n = 6	-
Mutation type			
Missense	4 (100)	5 (83.3)	1
G-M-Y	0 (0)	1 (16.7)	1
M-X-Y	4 (100)	3 (50.0)	0.2
Charged/Branched-chain AAs	4 (100)	3 (50.0)	0.2
Frameshift	0 (0)	1 (16.7)	1
Domain			
NC1	0 (0)	1 (16.7)	1
Triple-helical	4 (100)	5 (83.3)	1

G-M-Y indicates that the mutation is located at the amino acid position after the glycine residue in the glycine triplet sequence. G-X-M indicates that the mutation is located at amino acid position two after the glycine residue in the glycine triplet sequence. M-X-Y indicates that the mutation is located at the glycine position in the glycine triplet sequence. The UTR represents the untranslated region. NC1 represents the non-collagenous domain. AAs are amino acids. CNV is an abbreviation for copy number variant.

Supplementary Table S5. Comparison of clinical and imaging findings between the CA-positive and CA-negative groups.

	CA positive	CA negative	p-value
	n = 25	n = 51	
Age at examination, mean ± sd, NA	37.4 ± 13.9, 7	31.2 ± 19.4, 2	0.2183
Median [IQR]	35.5 [24,50]	31 [17, 46.5]	-
Age at stroke onset, mean ± sd, NA	32.3 ± 14.0, 13	30.2 ± 15.6, 26	0.8710
	33.5 [21, 42.5]	34 [17, 43.25]	-
Male, n (%), NA	8 (34.8), 2	26 (52.0), 1	0.2113
Risk factors			
HT, n (%), NA	1 (10),15	5 (17.9),23	1
Smoking, n (%), NA	1 (12.5),17	6 (50.0),39	0.1580

Family history			
Family history, non-SAH stroke n (%), NA	13 (61.9), 4	17 (40.5), 9	0.12
Family history, SAH n (%), NA	1 (5.0), 5	1 (2.6),13	1
Family history, CA, n (%), NA	6 (66.7),16	9 (50.0),33	0.6828
Region, n (%)			
Africa	0	1 (2.0)	
Asia	6 (24.0)	14 (27.5)	
Central/South America	1 (4)	2 (3.9)	
Europe	16 (64.0)	26 (51.0)	0.85586*
Middle East	0	2 (3.9)	
North America	2 (8.0)	5 (9.8)	
Undetermined	0	1 (2.0)	
De Novo mutation , n (%)	4 (16.0)	9 (17.6)	1
Stroke	15 (62.5), 1	29 (58.0), 1	0.8030
Ischemic stroke n (%), NA	8 (38.1), 4	15 (31.3), 3	0.5906
ICH n (%), NA	5 (22.7), 3	14 (29.8), 4	0.7731
SAH n (%), NA	0 (0), 4	1 (2.2), 5	1
Other stroke n (%), NA	0 (0), 4	0 (0), 5	1
Migraine, n (%), NA	4 (44.4),16	1 (6.3), 35	0.0403
Cognitive impairments/intellectual abnormality, n (%), NA	4 (40),15	16 (59.3),24	0.4597
RAT, n (%), NA	11 (64.7), 8	14 (58.3),27	0.7533
Muscle Cramp, n (%), NA	9 (60.0),10	10 (50.0),31	0.7338
Nephropathy, n (%), NA	10 (71.4),11	13 (52.0),26	0.3172
Seizure, n (%), NA	2 (28.6),18	11 (73.3),36	0.0743
Brain imaging findings			
Structure abnormality of CNS, n (%), NA	3 (27.3),14	15 (46.9),19	0.3090
Leukoencephalopathy, n (%), NA	20 (87.0), 2	34 (69.4), 2	0.1480
MBs, n (%), NA	10 (90.9), 14	19 (82.6), 28	1
Lacunar, n (%), NA	6 (60.0), 15	16 (69.6), 28	0.6960
Aneurysm, n (%), NA	25 (100), 0	-	-
ICA, n (%), NA	19 (86.4), 3	-	-
MCA, n (%), NA	1 (4.8), 4	-	-
Acom, n (%), NA	1 (4.8), 4	-	-
Pcom, n (%), NA	1 (4.8), 4	-	-
BA, n (%), NA	2 (9.5), 4	-	-
SCA, n (%), NA	1 (4.8), 4	-	-
Multiple, n (%), NA	12 (54.5), 3	-	-
The other large vessel abnormality, n(%), NA	7 (33.3), 4	8 (20.5), 12	0.3521
Stenosis/Occlusion, n (%), NA	3 (15.0), 5	3 (7.7), 12	0.3976
Dissection, n (%), NA	1 (4.8), 4	0 (0), 12	0.3500
Dolichoectasia, n (%), NA	3 (15.0), 5	2 (5.1), 12	0.3246
others, n (%), NA	1 (4.8), 4	4 (10.3), 12	0.6486

CA indicates cerebral aneurysm; HT, hypertension; SAH, subarachnoid hemorrhage; ICH, intracerebral hemorrhage; RAT, retinal artery tortuosity; CNS, central nervous system; MBs, microbleeds; MRA, magnetic resonance angiography; CTA, computed tomography angiography; ICA, internal carotid artery; MCA, middle cerebral artery; Acom, anterior communicating artery; Pcom, posterior communicating artery; BA, basilar artery; SCA, superior cerebellar artery; NA, not available. *Chi-squared tests.

Supplementary Figure S1. Flow diagram of included publications

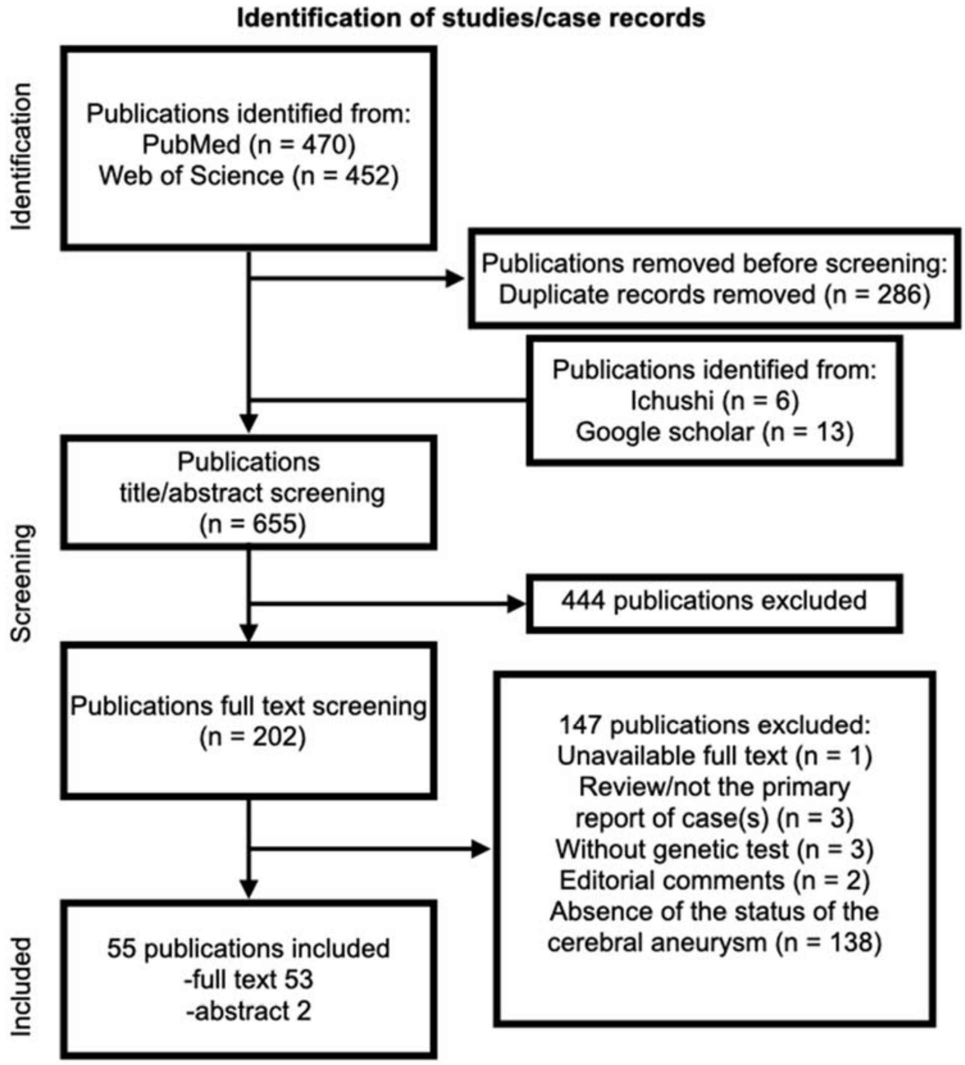

Supplement: Supplementary file 1 [file neurolint-16-00015-s001.zip › neurolint-2790730-supplementary.pdf]
